# Impact of a structured food sequence and mobile health monitoring on gestational diabetes outcomes: a clinical trial

**DOI:** 10.3389/fnut.2025.1562240

**Published:** 2025-07-28

**Authors:** Ria Murugesan, Shubhashree Thiruselvam, Kakithakara Vajravelu Leela, Abhishek Satheesan, K. Geetha, Mohan Ram, Janardanan Kumar

**Affiliations:** ^1^Department of Microbiology, SRM Medical College Hospital and Research Centre, SRM IST, Kattankulathur, Tamil Nadu, India; ^2^Department of Obstetrics and Gynecology, SRM Medical College Hospital and Research Centre, SRM IST, Kattankulathur, Tamil Nadu, India; ^3^Department of Clinical Nutrition and Dietetics, SRM Medical College Hospital and Research Centre, SRM IST, Kattankulathur, Tamil Nadu, India; ^4^Department of Medical Laboratory Technology, SRM Medical College Hospital and Research Centre, SRM IST, Kattankulathur, Tamil Nadu, India; ^5^Department of General Medicine, SRM Medical College Hospital and Research Centre, SRM IST, Kattankulathur, Tamil Nadu, India

**Keywords:** gestational diabetes mellitus, food order, mobile health application, blood glucose control, pregnancy outcomes

## Abstract

**Background:**

Gestational Diabetes Mellitus (GDM) is a common pregnancy complication that poses risks to both mother and baby. Dietary management is crucial in controlling blood glucose, and recent evidence suggests that the order in which food is consumed (food sequencing) can improve glycemic control. Mobile health (mHealth) tools also offer promising support for healthier eating habits and glucose monitoring. This study evaluates whether combining a structured food sequence with mHealth monitoring enhances outcomes in women with GDM.

**Methods:**

Fifty-four pregnant women diagnosed with GDM were randomized into an intervention group (*n* = 27) and a control group (*n* = 27). The intervention group followed a structured food order—fiber first, then protein, and carbohydrates last—and tracked their intake using a mobile health application (JotForm). The control group received standard antenatal care without food sequencing. Fasting and postprandial glucose, lipid profiles, hemoglobin, and pregnancy outcomes were measured at baseline, the end of the third trimester, and 4 weeks postpartum. Data analysis included paired and independent *t*-tests or non-parametric equivalents, with chi-square tests for categorical variables. A *p*-value <0.05 was considered statistically significant.

**Results:**

The intervention group showed significant reductions in 1-h and 2-h postprandial blood glucose levels (−8.41 mg/dL and −7.56 mg/dL respectively, both *p* < 0.001), decreased LDL cholesterol (−7.33 mg/dL, *p* < 0.001), increased HDL cholesterol (+6.15 mg/dL, *p* < 0.001), and improved hemoglobin levels. They also had more normal deliveries (13 vs. 10) and fewer cases of shoulder dystocia (2 vs. 5) compared to controls. Additionally, this group exhibited lower average birth weights, fewer NICU admissions, and reduced neonatal complications.

**Conclusion:**

Combining a structured food sequencing approach with mHealth dietary monitoring improved maternal glycemic control and pregnancy outcomes in women with GDM. This strategy shows promise for supporting dietary adherence and managing GDM effectively, warranting further research to validate its broader application.

**Clinical trial registration:**

https://ctri.nic.in/Clinicaltrials/main1.php?EncHid=93509.81482, identifier CTRI/2024/01/061220.

## 1 Introduction

Gestational Diabetes Mellitus (GDM) is a condition characterized by glucose intolerance during pregnancy, often due to insulin resistance induced by placental hormones. GDM increases the risk of maternal complications such as preeclampsia (hypertensive disorder), fetal macrosomia (excessive fetal growth), and the need for operative delivery (cesarean section). It can also lead to shoulder dystocia (difficult birth due to fetal shoulder impaction), higher rates of neonatal intensive care unit (NICU) admissions, and impaired neonatal metabolic regulation. Long-term, GDM elevates the risk of Type 2 Diabetes Mellitus (T2DM) in the mother, while offspring face a heightened risk of obesity and metabolic syndrome (1). Effective management of GDM is crucial to minimizing these complications and improving both immediate and long-term health outcomes. Recent advances have highlighted the importance of the gut microbiota–GDM axis in understanding GDM pathophysiology (2). Dysbiosis, or disruption of the gut microbial balance, has been implicated in promoting systemic inflammation, insulin resistance, and altered maternal-fetal metabolic signaling (3). Modulation of the maternal microbiota through diet is increasingly seen as a potential therapeutic strategy, reinforcing the relevance of dietary interventions in GDM management.

In parallel, the Developmental Origins of Health and Disease (DOHaD) framework emphasizes how in utero exposures, including maternal hyperglycemia, can induce long-lasting epigenetic changes in the fetus, influencing lifelong susceptibility to metabolic disorders (4). Thus, optimal glycemic control during pregnancy not only improves perinatal outcomes but may also reduce the risk of chronic diseases in the offspring (5).

While diet and exercise are central to managing GDM, ensuring adherence to dietary recommendations is often challenging due to behavioral, social, and logistical barriers. Mobile health (mHealth) technologies offer a promising solution by enabling real-time tracking, personalized feedback, and continuous engagement (6, 7). These digital tools have shown potential to improve adherence to lifestyle interventions and enhance communication between patients and healthcare providers (6).

Emerging research highlights that not only the types of food consumed but also the order in which foods are taken can significantly influence blood glucose levels (8). Specifically, consuming food in Order of fibre first, followed by protein, and carbohydrates last, has shown promising results in reducing postprandial blood glucose spikes (9). This strategy is based on the physiological processes of nutrient absorption—fibre and protein play essential roles in slowing the absorption of glucose into the bloodstream, helping to stabilize blood sugar levels after meals (10). Fibre, when consumed first, helps to slow the gastric emptying process, which in turn delays the absorption of glucose from carbohydrates. This helps prevent the rapid spikes in blood sugar that typically follow carbohydrate-rich meals. Protein further supports this by promoting the release of hormones, such as insulin and glucagon-like peptide-1 (GLP-1), which help in regulating blood sugar levels and slow down gastric emptying (11). It also helps maintain satiety, preventing overeating, and contributes to a steadier glucose response. When consumed after fibre, protein continues to slow down digestion and enhance satiety, further stabilizing blood glucose levels (12, 13). Carbohydrates, when consumed last, are then absorbed more gradually, preventing a sharp increase in blood glucose. This sequencing of macronutrients (fibre, protein, carbohydrates) can significantly improve postprandial blood glucose control, which is particularly important for women with GDM who need to manage their blood sugar levels without the use of insulin or other medications. The link between Food Order and improved pregnancy outcomes, such as reduced risk of macrosomia, fewer NICU admissions, and fewer complications, lies in the stabilization of blood glucose levels (14, 15). By reducing postprandial blood sugar spikes, food sequencing can help prevent excessive fetal growth (macrosomia) and the associated complications, such as shoulder dystocia or the need for a cesarean section. Stabilized blood glucose also decreases the likelihood of neonatal hypoglycemia, leading to fewer NICU admissions (16). Further, maintaining more consistent glucose levels through food sequencing supports the overall maternal health, reducing the risk of complications like preeclampsia and promoting normal deliveries (17). Therefore, this structured dietary intervention not only aids in the management of blood glucose but also has a direct impact on both maternal and fetal health outcomes, including birth weight and the incidence of complications during delivery. Mobile health applications have become a vital tool in modern healthcare, especially for managing chronic conditions and pregnancy-related complications (18). With the widespread use of smartphones, these apps provide easy access to health tracking, personalized advice, and feedback, making them an essential part of disease management. In the context of GDM, mobile health apps help users to monitor and track meals, adhere to dietary guidelines, and offer convenience and continuous support (6, 7). They enhance their communication with healthcare providers, enable personalized care, and improve adherence to treatment plans. Additionally, these apps reduce healthcare costs and provide long-term benefits, such as preventing the development of T2DM (19). The integration of mobile health applications in managing GDM offers a convenient, cost-effective solution for improving maternal and fetal health outcomes by facilitating ongoing monitoring and personalized interventions (20). The novelty of this study lies in its dual intervention - a structured dietary approach that emphasizes food sequencing and is supported by the JotForm mobile health application. Through this app, participants can track their food intake and receive feedback, adhering to the recommended Food Order. While mobile health technologies have been explored for managing chronic conditions like T2DM, their use in GDM, particularly for dietary monitoring and food sequencing, is still underexplored. This study aims to investigate how this combined approach can improve dietary adherence, enhance maternal and fetal health outcomes, and offer a more personalized solution in managing GDM. In addition to addressing immediate pregnancy outcomes, this study also aims to explore the potential long-term benefits of dietary interventions in GDM. Specifically, the study will examine how early dietary modifications may reduce the future risk of developing T2DM in women who have had GDM. This research is significant because it explores an innovative, non-pharmacological intervention—food sequencing—paired with mobile health technology to improve GDM management. By focusing on both short-term pregnancy outcomes and long-term health risks, this study holds the potential to provide an effective, sustainable, and accessible approach for managing GDM, ultimately benefiting both maternal and fetal health.

## 2 Methodology

This study was conducted from February 2024 to November 2024 at SRM Medical College Hospital and Research Centre, Chennai, India.

### 2.1 Ethical considerations

The study was registered in the Clinical Trials Registry-India CTRI/2024/01/061220^1^ after being approved by the institutional ethical committees (Institutional Ethical Clearance Number: 8707/IEC/2023). Informed consent was obtained from all participants.

### 2.2 Sample size calculation

The sample size was calculated using G*Power software with a two-tailed test, assuming a type I error of 5% (*α* = 0.05), type II error of 20% (power = 80%), and an effect size of 0.6 based on previous studies assessing dietary interventions in GDM (21, 22).

### 2.3 Study participants

**Figure 1:**
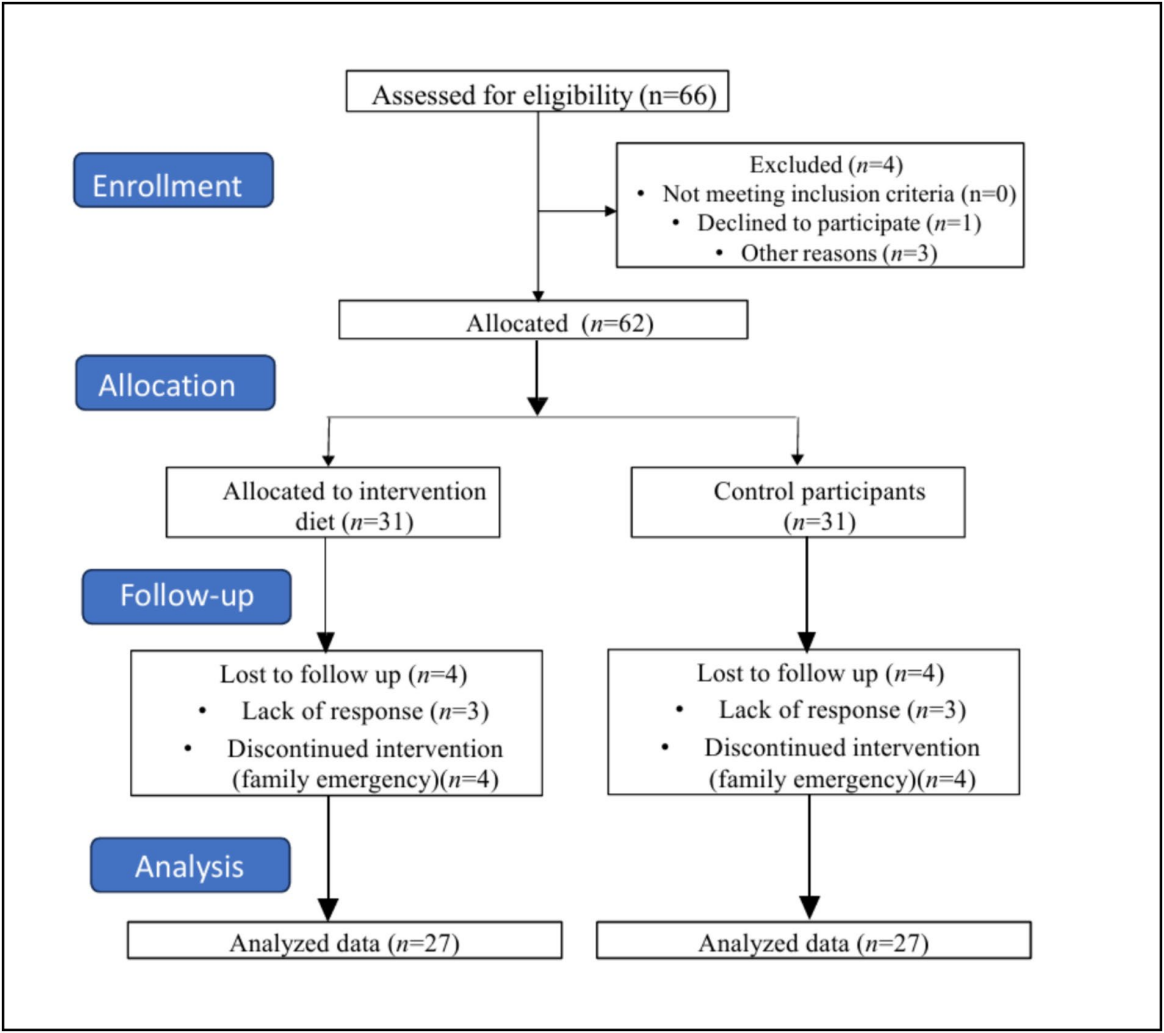
CONSORT flow diagram.

Fifty-four women diagnosed with GDM, willing to participate, were recruited from an obstetric OPD clinic. The participants were divided into two groups: the intervention group (*n* = 27), which followed a structured Food Order (fibre first, followed by protein, and carbohydrates last), and the control group (*n* = 27), which received routine care (Table 1). At baseline, no significant variations in biochemical measures were observed between the Food Order and Control group, which sets a solid foundation for evaluating the effects of the Food Order intervention. The study protocol was thoroughly explained to all participants, and informed consent was obtained before the start of the study. An information sheet detailing the intervention was provided to each participant and carefully reviewed to ensure a clear understanding of the study procedure (Figure 1).

**Table 1:** Baseline characteristics of study participants.

GDM (54)	Food order group	Control group	*p-*value
Age (Years)	26.93 ± 2.51	27.26 ± 2.78	0.646
Gestational week	26.48 ± 0.89	26.41 ± 0.93	0.767
Height (cm)	156.59 ± 4.99	156.81 ± 4.30	0.862
Weight (kg)	73.54 ± 9.94	72.89 ± 11.13	0.82
BMI (kg/m^2^)	29.98 ± 3.28	30.15 ± 3.72	0.856
HbA1c (%)	5.730 ± 0.43	5.77 ± 0.41	0.701
Systolic BP (mmHg)	114.44 ± 10.79	113.30 ± 11.54	0.707
Diastolic BP (mmHg)	74.33 ± 7.37	73.11 ± 7.29	0.551
FBG (mg/dL)	100.74 ± 5.193	100.85 ± 4.928	0.936
1 h PPBG (mg/dL)	155.63 ± 6.907	153.78 ± 6.320	0.309
2 h PPBG (mg/dL)	126.63 ± 4.413	127.44 ± 4.742	0.516
Hb (g/dL)	10.303 ± 0.740	10.588 ± 0.707	0.154
Total Cholesterol (mg/dL)	221.37 ± 19.881	222.89 ± 19.041	0.776
LDL-c (mg/dL)	144.96 ± 5.338	147.04 ± 5.110	0.151
HDL-c (mg/dL)	45.56 ± 5.774	45.07 ± 6.170	0.768
Triglycerides (mg/dL)	179.22 ± 52.657	176.63 ± 40.482	0.841

Data are represented as mean ± SD, obtained from an independent Student *t*-test for the between-group comparisons. cm, centimeter; kg, kilogram; mmHg, millimeters of mercury, FBG, fasting blood glucose; PPBG, postprandial blood glucose; HbA1c, glycated hemoglobin; LDL-c, low-density lipoprotein cholesterol; HDL-c, high-density lipoprotein cholesterol; VLDL-c, very-low-density lipoprotein cholesterol; mg/dL, milligrams per deciliter.

### 2.4 Study criteria

#### 2.4.1 Inclusion criteria

GDM on lifestyle modification (diet + exercise), aged 18–40 years, gestational weeks between 24 and 28 weeks, signed an informed consent, and comfortable using a mobile health application for their health management.

#### 2.4.2 Exclusion criteria

Women with pre-existing type 1 or type 2 diabetes, use of insulin therapy or any other blood glucose-lowering drugs (BGLDs), below 24 weeks of gestation, early-onset of GDM, pre-term delivery, women unable to comply with study procedures, any severe medical conditions, uncontrolled hypertension, severe food allergies or intolerances from specific dietary intervention.

### 2.5 Study design

**Figure 2:**
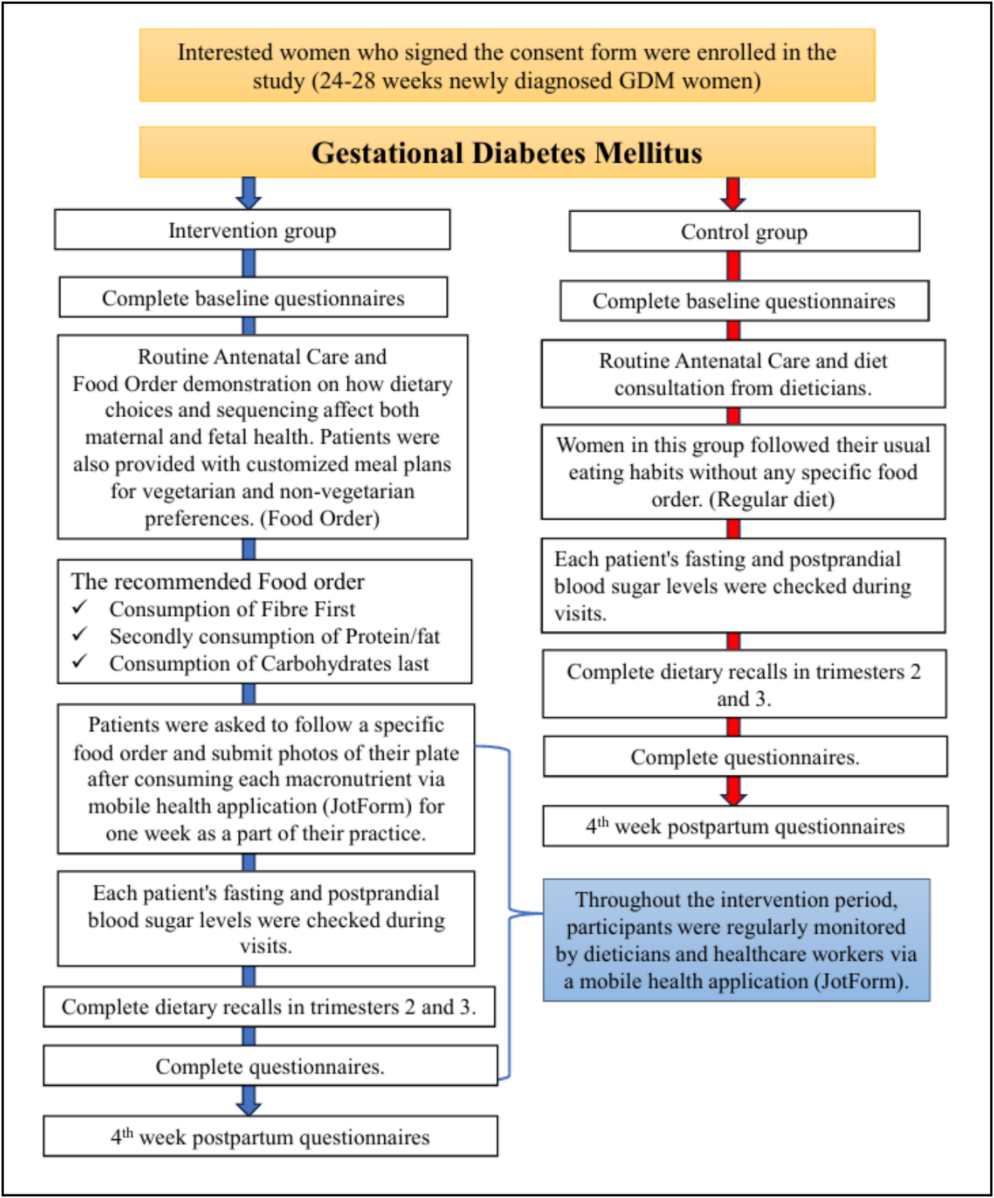
Study flowchart.

This was an open-label, interventional study conducted to evaluate the impact of a structured Food Order dietary intervention on glycemic control in women with GDM. Group allocation was based on convenience sampling, as randomization and blinding were not feasible due to real-world clinical constraints. This non-randomized approach may introduce selection and observation bias, which is acknowledged as a study limitation. Efforts were made to compare baseline characteristics to assess group comparability (Figure 2).

#### 2.5.1 Intervention group

The intervention group followed a well-defined protocol designed to enhance glycemic control in pregnant women with GDM. Initially, baseline evaluations were conducted, which included questionnaires to assess dietary habits, lifestyle, and health status, as well as clinical measurements such as blood glucose levels (fasting, 1-h, and 2-h), Hb (hemoglobin), BMI (Body Mass Index), lipid profile, HbA1c (Hemoglobin A1c), and blood pressure. Subsequently, participants received antenatal care, including regular check-ups and blood tests. The central element of the intervention was a structured dietary plan with a specific food sequence: fibre was consumed first, followed by proteins and fats, and carbohydrates were consumed last. Tailored meal plans were provided for both vegetarian and non-vegetarian participants, who were instructed to follow this regimen. To ensure adherence, participants used a mobile health application (JotForm) to upload photos of their meals after consuming each macronutrient. This allowed dietitians to monitor compliance, offer feedback, and address any concerns. The application, available on both Android and iOS platforms, featured an intuitive interface, enabling easy meal logging and communication with dietitians. It also collected data on meal adherence, which dietitians reviewed regularly to guide throughout the intervention period. Participants were advised to maintain their normal physical activity levels, such as walking, low-impact aerobics, without overexerting themselves throughout the study. Detailed dietary recalls were conducted during the second and third trimesters to capture comprehensive dietary intake information. Follow-up measurements were taken at 36–40 weeks, just before delivery, to compare improvements in food sequence adherence and other clinical parameters from baseline measurements. This allowed for a comparison of the effectiveness of the dietary and lifestyle interventions in managing GDM. Mode of delivery and any complications during delivery were also noted. A 4th week postpartum questionnaire was administered at the end of the study to evaluate the long-term effects of the dietary intervention. Meal adherence was monitored using self-reported photo uploads via the mobile application, which may be subject to bias. The specific diet plans followed during the intervention period, including the detailed composition of fibre, protein sources, and carbohydrate foods, are provided in the Supplementary material for both the second and third trimesters. Throughout the intervention, participants were continuously monitored by dietitians and healthcare providers, both via the mobile application and regular visits, to ensure adherence to the dietary plan and offer ongoing support.

#### 2.5.2 Control group

The control group received routine antenatal care following the standard practice for managing GDM. Baseline evaluations were conducted, which included questionnaires to assess dietary habits, lifestyle, and health status, as well as clinical measurements such as fasting and postprandial blood glucose levels (1 h, 2 h), Hb, BMI, blood pressure, lipid profile, and HbA1c. Participants continued with regular antenatal check-ups, including routine blood tests, as part of standard care. Consultation with a dietitian was provided, offering general advice on healthy eating and glucose management, but without any specific dietary orders. The control group was not required to use mobile tracking tools or follow any specific food sequence diet plan guidelines. During the second and third trimesters, participants underwent dietary recalls to monitor their nutritional intake during their visit. Follow-up measurements were taken at 36–40 weeks, just before delivery, to assess any changes in food sequence adherence and clinical parameters from the baseline. Mode of delivery and any complications during delivery were also noted. Additionally, a 4^th^ week postpartum questionnaire was taken to evaluate the participants’ health and post-delivery. This enabled the comparison of the effectiveness of the dietary and lifestyle interventions in the intervention group (Food Order) against the standard care provided to the control group (without Food Order) in managing GDM.

### 2.6 Mobile health application for dietary recall tracking

The Jotform mobile health application was used to track dietary recall and monitor participants’ adherence to the food-ordering regimen. Jotform, a popular platform for creating forms in clinical research, is known for its user-friendly design and standardization in data collection. The app’s front-end interface is built with JavaScript (React.js), while the backend is supported by PHP and MySQL for data processing and storage. It utilizes Amazon Web Services (AWS) for cloud hosting, ensuring both scalability and reliability. This mobile application has been widely used in recent clinical studies for dietary tracking, proving its effectiveness and ease of use (23–25). The application enabled participants to log meals by selecting meal types (such as breakfast, lunch, and dinner), uploading meal photos, and reporting macronutrient information. During an initial onboarding session, participants were trained on how to log their app and submit photos of their plates before and after eating. Healthcare workers and dietitians were also trained to review the meal logs and provide feedback to support compliance with the dietary intervention. The reliance on self-logging and photograph-based meal tracking is noted as a limitation due to the potential for reporting bias.

### 2.7 Measurement of outcomes

Outcomes were measured by assessing blood glucose levels at fasting, 1 h postprandial, and 2 h postprandial to evaluate the impact of dietary interventions on glycemic control. Blood glucose was measured using the glucose oxidase enzymatic method, a widely trusted technique. The Beckman Coulter DxC 700 AU analyzer was used to ensure accurate and consistent results. Cholesterol levels, including total cholesterol, LDL-c (Low-Density Lipoprotein Cholesterol), HDL-c (High-Density Lipoprotein), and triglycerides, were assessed with enzymatic assays. HbA1c, a key marker for long-term glycemic control, was measured using high-performance liquid chromatography (HPLC), which effectively separates and quantifies HbA1c. BMI was calculated using the formula: weight (kg) / height (m^2^), while blood pressure was measured using a calibrated sphygmomanometer after a five-minute rest in a seated position, with two readings taken to ensure accuracy. These measurements provided essential data for evaluating the effects of dietary interventions on glycemic control and other critical health markers related to gestational diabetes.

### 2.8 Statistical analysis

Data were analyzed using paired *t*-tests for within-group comparisons and independent *t*-tests for between-group comparisons, after assessing the assumptions of normality and homogeneity of variances. Normality was evaluated using the Shapiro–Wilk test, and Levene’s test was applied to assess equality of variances. Chi-square tests were used for categorical variables. Given the small sample size, basic statistical tests were selected to maintain interpretability and reduce the risk of overfitting. However, we acknowledge that future studies with larger and more diverse cohorts should incorporate multivariate regression models to adjust for potential confounding variables and to enhance the robustness of findings. Statistical significance was set at *p* < 0.05. Data are presented as mean ± standard deviation (SD).

## 3 Results

### 3.1 Comparison of metabolic and lipid profile changes in the food order and control groups before and after intervention

The table presents the changes in various metabolic and lipid profile parameters in the Food Order and Control group, measured before and after the intervention (between 36 and 40 weeks). The intervention group demonstrated potential improvements in 1-h postprandial blood glucose (1 h PPBG), 2-h postprandial blood glucose (2 h PPBG), hemoglobin (Hb), and LDL-c. Specifically, the 1 h PPBG decreased by 8.41 mg/dL (*p* < 0.001), the 2 h PPBG decreased by 7.56 mg/dL (*p* < 0.001), Hb increased by 1.20 g/dL (*p* < 0.001), and LDL-c decreased by 7.33 mg/dL (*p* < 0.001). Additionally, HDL-c increased by 6.15 mg/dL (*p* < 0.001). However, fasting blood glucose (FBG) and triglycerides did not show significant changes in this group (*p* = 0.101 and *p* = 0.283, respectively), and total cholesterol also showed no significant change (*p* = 0.83). In the Control group, significant changes were observed in 1 h PPBG, 2 h PPBG, and LDL-c. The 1 h PPBG increased by 1.48 mg/dL (*p* = 0.100), while the 2 h PPBG increased by 3.04 mg/dL (*p* = 0.002), and LDL-c decreased by 2.96 mg/dL (*p* = 0.003). Hemoglobin in the Control group increased by 0.81 g/dL (*p* < 0.001), similar to the Food Order group. However, FBG, total cholesterol, HDL-c, and triglycerides did not show statistically significant changes (*p* = 0.466, *p* = 0.306, *p* = 0.671, and *p* = 0.691, respectively). However, there were no significant differences between the two groups in fasting blood glucose, total cholesterol, and triglycerides. Inter-group comparison (*p*^b^ values) revealed that the Food Order group showed significant improvements in 1 h PPBG, 2 h PPBG, Hb, and LDL-c, as compared to the Control group, which exhibited significant changes in 2 h PPBG and LDL-c. These findings indicate favorable metabolic changes in the Food Order group compared to the control group (Table 2).

**Table 2:** Within-group and between-group comparisons of metabolic profiles.

Parameters	Food order group				Control group				
	Before	After	Change	*p* ^a^	Before	After	Change	*p* ^a^	*p* ^b^
FBG (mg/dL)	100.74 ± 5.193	98.74 ± 3.392	−2.001 ± 6.120	0.101	100.85 ± 4.928	101.93 ± 5.935	1.0741 ± 7.549	0.466	0.106
1 h PPBG (mg/dL)	155.63 ± 6.907	147.22 ± 4.353	−8.407 ± 6.749	<0.001*	153.78 ± 6.320	155.26 ± 4.486	1.481 ± 4.518	0.100	<0.001*
2 h PPBG (mg/dL)	126.63 ± 4.413	119.07 ± 2.814	−7.555 ± 5.1015	<0.001*	127.44 ± 4.742	130.48 ± 4.870	3.0370 ± 4.552	0.002	<0.001*
Hb (g/dL)	10.303 ± 0. 740	11.507 ± 0. 802	1.203 ± 1.0825	<0.001*	10.588 ± 0. 707	11.400 ± 0. 643	0.811 ± 1.005	<0.001*	0.173
Total cholesterol (mg/dL)	221.37 ± 19.881	213.56 ± 10.653	−7.814 ± 22.530	0.83	222.89 ± 19.041	220.19 ± 16.820	−2.703 ± 13.458	0. 306	0.316
LDL-c (mg/dL)	144.96 ± 5.338	137.63 ± 5.759	−7.333 ± 5.929	<0.001*	147.04 ± 5.110	144.07 ± 6.759	−2.963 ± 4.727	0.003	0.004
HDL-c (mg/dL)	45.56 ± 5.774	51.70 ± 6.145	6.148 ± 7.129	<0.001*	45.07 ± 6.170	44.48 ± 6.247	−0.592 ± 7.158	0.671	<0.001*
Triglycerides (mg/dL)	179.22 ± 52.657	171.04 ± 40.412	−8.185 ± 38.771	0.283	176.63 ± 40.482	180.33 ± 38.731	3.703 ± 47.828	0.691	0.320

Data are represented as mean ± SD. ^a^ Obtained from a paired *t*-test for the within-group comparisons. ^b^ Obtained from an independent Student *t*-test for the between-group comparisons. FBG, fasting blood glucose; PPBG, postprandial blood glucose; HbA1c, glycated hemoglobin; LDL-c, low-density lipoprotein cholesterol; HDL-c, high-density lipoprotein cholesterol; mg/dL, milligrams per decilitre. Statistically significant values are denoted as “*”.

### 3.2 Obstetric outcomes and postpartum complications in the food order and control groups

Obstetric outcomes and postpartum complications were compared between the Food Order and Control groups. While no statistically significant differences were observed, small effect sizes across multiple outcomes suggest trends favoring the intervention group. In terms of mode of delivery, 13 participants (48.1%) in the Food Order group had normal vaginal deliveries, 11 (40.7%) underwent cesarean sections, and 3 (11.1%) had instrumental deliveries. In the Control group, 10 participants (37.0%) had normal deliveries, 12 (44.4%) underwent cesarean sections, and 5 (18.5%) had instrumental deliveries. The overall distribution did not differ significantly between groups (*p* = 0.627), with a very small effect size (0.13). Shoulder dystocia occurred in 2 participants (7.4%) in the Food Order group and 5 participants (18.5%) in the Control group. Although not statistically significant (*p* = 0.418), the effect size (0.11) indicates a small effect favoring the intervention group. NICU admission was required in 1 case (3.7%) in the Food Order group and 3 cases (11.1%) in the Control group (*p* = 0.603; effect size = 0.07), representing a negligible effect. Breathlessness in newborns was reported in 2 cases (7.4%) in the Food Order group and 4 cases (14.8%) in the Control group. Although this variable was not analyzed separately due to low frequency, the trend favored the Food Order group. By 4 weeks postpartum, new-onset T2DM was diagnosed in 2 participants (7.4%) in the Food Order group and 6 participants (22.2%) in the Control group. This difference was not statistically significant (*p* = 0.250), but the effect size (0.16) suggests a small potential benefit associated with the food sequencing intervention. In summary, although no outcomes reached statistical significance, small effect sizes observed across several parameters indicate a potentially favorable trend in obstetric and postpartum outcomes in the Food Order group compared to the Control group (Table 3).

**Table 3:** Statistical analysis of clinical outcomes between food order and control groups.

Outcome	Food order group (*n* = 27)	Control group (*n* = 27)	*p-*value	Effect size
Mode of delivery				
Normal	13 (48.1%)	10 (37.0%)	0.627	0.132
Cesarean	11 (40.7%)	12 (44.4%)		
Instrumental	3 (11.1%)	5 (18.5%)		
Shoulder dystocia	2 (7.4%)	5 (18.5%)	0.418	0.110
NICU admission	1 (3.7%)	3 (11.1%)	0.603	0.071
T2DM at 4 weeks postpartum	2 (7.4%)	6 (22.2%)	0.250	0.156

Data are presented as frequency (percentage), *p*-values derived from Chi-square tests as appropriate. Effect size is based on Cramér’s V values, indicating the strength of association between groups.

### 3.3 Birth weight distribution and statistical comparison

**Figure 3:**
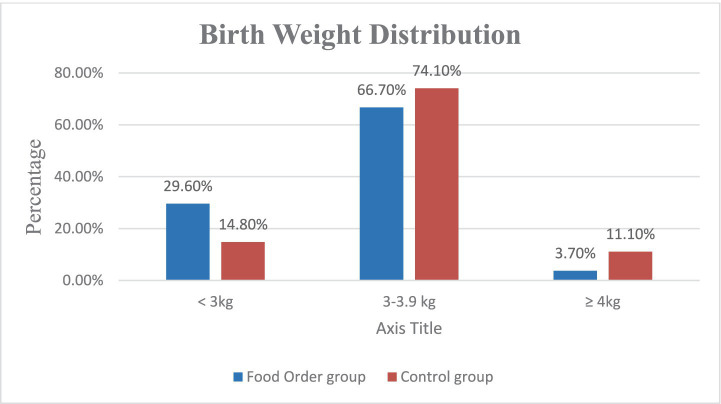
Comparison of birth weight distribution between food order and control groups.

Birth weight data were analysed to compare fetal growth outcomes between the Food Order and Control groups. The mean birth weight in the Food Order group was 3.14 ± 0.34 kg, while the Control group had a higher mean birth weight of 3.32 ± 0.41 kg. Although this difference did not reach statistical significance (*p* = 0.068), it suggests a trend toward lower average birth weight in the intervention group. Further categorization of birth weights revealed that 29.6% of neonates in the Food Order group weighed <3.0 kg compared to 14.8% in the Control group. In contrast, the incidence of macrosomia (birth weight ≥4.0 kg) was lower in the Food Order group (3.7%) compared to the Control group (11.1%). The majority of births in both groups were within the 3.0–3.9 kg range (Table 4). These findings suggest a potentially favorable effect of the dietary intervention on fetal growth regulation, likely mediated by improved glycemic control in the Food Order group (Figure 3).

**Table 4:** Birth weight distribution between groups.

Birth weight category	Food order group (*n* = 27)	Control group (*n* = 27)	*p*-value
Mean ± SD (kg)	3.14 ± 0.34	3.32 ± 0.41	0.068^1^
<3.0 kg	8 (29.6%)	4 (14.8%)	
3.0–3.9 kg	18 (66.7%)	20 (74.1%)	
≥4.0 kg (Macrosomia)	1 (3.7%)	3 (11.1%)	

1 Mean comparison via independent *t*-test; categorical comparisons via Chi-square test. Statistical comparisons for categorical data were conducted using the Chi-square test.

## 4 Discussion

**Figure 4:**
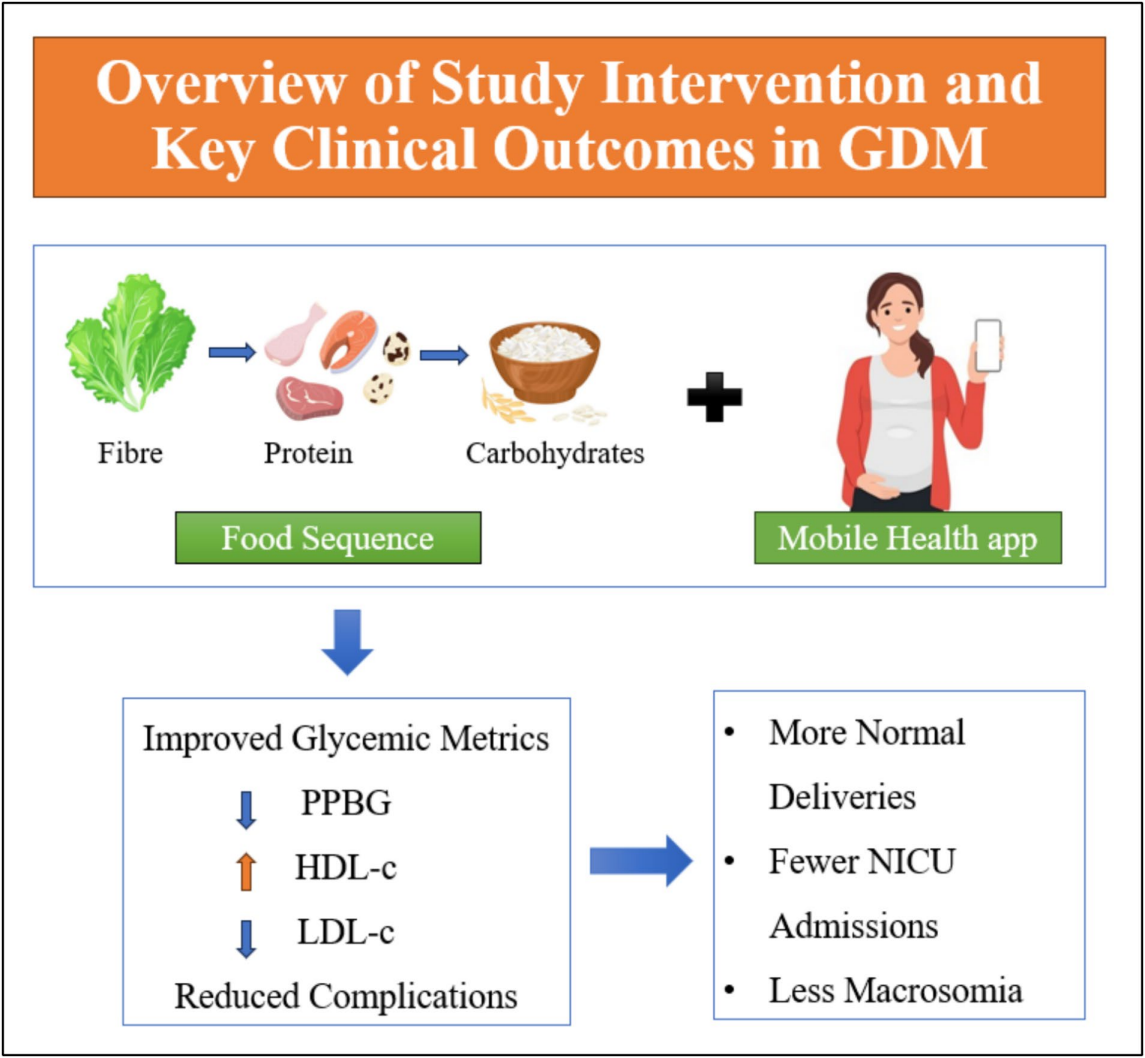
Overview of study intervention and key clinical outcomes in GDM.

This study evaluated the effects of a dietary intervention that used a structured Food Order strategy supported by mHealth on maternal and fetal outcomes in women with GDM. The results indicate that the intervention group showed significant improvements in postprandial blood glucose levels, lipid profiles, and hemoglobin compared to the control group. Moreover, the intervention group experienced better delivery outcomes, with a higher incidence of normal deliveries and a lower incidence of shoulder dystocia. These findings suggest that the use of mHealth applications, in combination with structured dietary interventions, may offer potential benefits for women with GDM, contributing to better maternal and fetal health. Furthermore, our results suggest that the sequence in which food components are consumed may influence birth weight. The Food Order group showed a lower mean birth weight (3.14 ± 0.34 kg) compared to the Control group (3.32 ± 0.41 kg), alongside a reduced rate of macrosomia (≥4.0 kg: 3.7% vs. 11.1%). Although the differences were not statistically significant, the trend supports the hypothesis that improved glycemic control through food sequencing may regulate fetal growth and reduce the risk of excessive birth weight. It is important to acknowledge several potential confounding variables that could have influenced the outcomes observed in this study. Physical activity levels, for instance, were not objectively measured and may have varied between participants, potentially impacting glycemic control and pregnancy outcomes. Similarly, dietary adherence, while supported through the mHealth application, was self-reported and could have introduced reporting bias or inconsistencies in compliance. Differences in educational status or health literacy may have influenced participants’ understanding and implementation of the dietary instructions. Moreover, socio-economic status (e.g., income, occupation, access to healthcare, or food quality) could have affected both intervention adherence and general health outcomes, although such variables were not formally controlled for in the current analysis. These factors represent important considerations when interpreting the findings and should be addressed in future studies through stratified analysis or more comprehensive baseline assessments. This study offers mechanistic insight into how structured food sequencing may influence postprandial hormonal responses, particularly insulin and GLP-1. Consuming fibre and protein first has been shown to delay gastric emptying and attenuate glucose absorption, which enhances the early secretion of GLP-1, a key incretin hormone that stimulates insulin release and suppresses glucagon. This hormonal modulation likely contributed to the observed reductions in postprandial glucose spikes, improved lipid profiles, and enhanced overall glycemic control seen in the intervention group (9, 10, 17). The novelty of this study lies in its integration of a simple, behaviorally anchored dietary strategy with mobile health support, specifically targeted at women with GDM. Unlike prior studies focusing on low-glycemic or general dietary advice, our structured food order intervention suggests promising improvements, not only in metabolic parameters but also in maternal and neonatal outcomes, including lower rates of shoulder dystocia, macrosomia, NICU admissions, and postpartum development. These findings underscore the dual hormonal and clinical benefits of food sequencing, positioning it as a practical, low-cost, and scalable strategy for improving outcomes in GDM, especially in resource-limited settings where dietary monitoring is difficult (Figure 4).

Several studies have explored interventions, including dietary strategies and mHealth application technologies, to improve glycemic control and pregnancy outcomes in women with GDM. The following comparison highlights how our study aligns with and supports previous research findings in this field. In H. Guo et al. (26) study, a mobile health intervention was used to improve glycemic control, reduce weight gain, and enhance compliance in pregnant women with GDM. Their findings revealed that the mHealth group demonstrated significantly improved compliance (83.3%) compared to the control group (70.4%), with reduced weight gain (3.2 ± 0.8 kg vs. 4.8 ± 0.7 kg, *p* < 0.001) and better glycemic control, including lower levels of HbA1c before delivery (4.7 ± 0.2% vs. 5.3 ± 0.3%, *p* < 0.001) (26). In our study, the intervention group also demonstrated significant improvements in glycemic control, with reductions in 1-h postprandial blood glucose (1 h PPBG: −8.41 mg/dL, *p* < 0.001) and 2-h postprandial blood glucose (2 h PPBG: −7.56 mg/dL, *p* < 0.001). Our intervention group also had lower birth weights and fewer complications like shoulder dystocia and NICU admissions, mirroring Guo et al.’s findings on the positive impact of mobile health interventions on both maternal health and pregnancy outcomes. The study by Meloncelli et al. (27) focused on the effects of a gut health-promoting diet, emphasizing prebiotics, fibre, and fermented foods. While their study specifically aimed to prevent GDM by improving gut microbiota and metabolic health, their findings also highlight the importance of dietary interventions. Although their results on glucose metabolism are still being evaluated, they suggest that dietary modifications can positively affect metabolic health and potentially prevent GDM. In comparison, our study focused on food sequencing, with fibre being consumed first, followed by protein and carbohydrates. This structured approach led to significant improvements in blood glucose levels and lipid profiles in our intervention group, including a notable increase in HDL cholesterol (+6.15 mg/dL, *p* < 0.001) and a decrease in LDL cholesterol (−7.33 mg/dL, *p* < 0.001), emphasizing the importance of dietary strategies in managing glucose metabolism. In Wen-Jun Ma et al. (28) randomized controlled trial on intensive low-glycemic-load (Low-GL) diets, they found significant improvements in glycemic control, with reductions in fasting plasma glucose (−0.33 mmol/L, *p* < 0.01) and 2-h postprandial glucose (−2.98 mmol/L, *p* < 0.01). Their study also reported a decrease in total cholesterol (0.12 mmol/L vs. 0.23 mmol/L, *p* < 0.05) and a lower increase in triglycerides (0.41 mmol/L vs. 0.56 mmol/L, *p* < 0.05). In our study, the intervention group showed a similar pattern, with significant reductions in 1-h and 2-h postprandial blood glucose levels and improvements in lipid profiles, such as a decrease in LDL cholesterol (−7.33 mg/dL, *p* < 0.001) and an increase in HDL cholesterol (+6.15 mg/dL, *p* < 0.001). Both studies suggest that dietary interventions focused on controlling the glycemic load can lead to better glycemic control and improved lipid metabolism. Yogev et al. (29) study on the relationship between blood glucose control and pregnancy complications concluded that strict control of blood glucose can reduce the rates of complications like macrosomia and shoulder dystocia. Our findings align with this conclusion, as the intervention group in our study had a lower incidence of shoulder dystocia (2 vs. 5 cases) and a higher rate of normal deliveries (13 vs. 10 cases). Additionally, we observed fewer NICU admissions in the intervention group (1 vs. 3) and the distribution of birth weights further supports this finding, as only one neonate (3.7%) in the Food Order group met the macrosomia threshold (≥4.0 kg), compared to three neonates (11.1%) in the Control group. This trend may reflect tighter glycemic control during pregnancy, which is known to reduce the risk of macrosomia and related complications. Crowther et al. (30) investigated the impact of tighter glycemic control on maternal and perinatal morbidity, concluding that achieving strict glycemic targets can significantly reduce complications like macrosomia, shoulder dystocia, and preeclampsia. Our study supports this conclusion, as we observed that the intervention group, with better blood glucose control, had fewer cases of shoulder dystocia, NICU admissions, and breathlessness compared to the control group. Additionally, the intervention group demonstrated a lower incidence of developing Type 2 diabetes by the fourth postpartum week (7.4% vs. 22.2% in the Control group), reinforcing the benefits of good glycemic control during pregnancy. Importantly, beyond statistical significance, several outcomes in this study hold notable clinical relevance. The reduction in shoulder dystocia cases (2 vs. 5) and NICU admissions (1 vs. 3) in the intervention group reflects tangible improvements in neonatal safety and resource utilization. Lower birth weights, with 70.4% of newborns in the intervention group weighing <3 kg compared to 14.8% in the control group, may indicate a reduction in macrosomia risk, a major factor in delivery complications. While these numbers are small due to sample size, they are consistent with broader clinical observations that tighter glycemic control can lead to improved delivery outcomes. Similarly, the lower incidence of postpartum development of T2DM (7.4% vs. 22.2%) in the intervention group suggests a potential long-term benefit for maternal metabolic health. These outcomes are not only statistically significant but also clinically meaningful, with implications for obstetric decision-making, postpartum care, and neonatal health planning. Our study findings align with the results from these studies, reinforcing the role of structured dietary interventions and mobile health technologies in improving glycemic control, weight management, and pregnancy outcomes in women with GDM. Like Guo et al., we demonstrated that mobile health tools can support better compliance and reduce complications, while our dietary intervention echoes the effectiveness of other nutritional approaches, such as those explored by Meloncelli et al. and Wen-Jun Ma et al. The positive outcomes seen in both maternal and fetal health further highlight the significance of glycemic control, as supported by Yogev et al. and Crowther et al. These studies collectively provide strong evidence for the effectiveness of dietary strategies and mobile health interventions in managing GDM and improving pregnancy outcomes. Future studies with larger sample sizes, objective monitoring of physical activity and dietary adherence, and stratification by educational and socio-economic status are warranted to validate and generalize these findings.

### 4.1 Limitations of the study

This study has some limitations that should be considered when interpreting the results. First, the relatively small sample size (*n* = 27 per group) limits the statistical power and generalizability of the findings, despite the observation of statistically significant differences. Second, the study employed an open-label design without randomization or blinding, which may introduce observation and reporting biases. The lack of random assignment to groups and the absence of blinding in both participants and investigators could have influenced adherence and outcome assessments. A more rigorous randomization process would strengthen the validity of future studies. Third, dietary adherence was self-reported via meal photographs and food logging through a mobile health (mHealth) application (JotForm), which may be subject to recall bias, underreporting, or misreporting, potentially affecting data accuracy. Fourth, the short-term nature of the follow-up, limited to the 4th week postpartum, restricts our ability to evaluate the long-term sustainability and clinical impact of the dietary intervention on both maternal and neonatal outcomes. Lastly, other confounding factors such as physical activity levels, psychosocial influences, and socio-economic status were not monitored or adjusted for in the analysis. These variables could have influenced the glycemic outcomes and should be considered in future studies to provide a more comprehensive understanding of the intervention’s effectiveness.

## 5 Conclusion

This study suggests that a structured food sequencing strategy, supported by mobile health monitoring, may potentially improve glycemic control and key maternal outcomes in women with gestational diabetes mellitus. The intervention not only enhanced postprandial glucose and lipid profiles but also contributed to better delivery outcomes, highlighting the synergistic benefits of combining dietary behavior modification with digital health tools. These findings highlight the potential of integrating personalized nutrition and technology-driven support as a practical, scalable approach in managing GDM. Despite limitations in sample size and study design, the observed improvements in glycemic markers and delivery outcomes underscore the promise of this low-cost, practical strategy as an adjunct to conventional management of GDM. These encouraging findings warrant larger, randomized controlled trials to validate the efficacy and long-term benefits of food sequencing interventions across diverse populations. Overall, this work advances understanding of dietary in metabolic control during pregnancy and highlights the value of combining nutritional strategies with digital health platforms.

## Data Availability

The original contributions presented in the study are included in the article/Supplementary material, further inquiries can be directed to the corresponding author.

## Footnotes

^1^ https://ctri.nic.in/Clinicaltrials/main1.php?EncHid=93509.81482
