# Supplementary material for: Impact of a structured food sequence and mobile health monitoring on gestational diabetes outcomes: a clinical trial
Source: Front Nutr. 2025 Jul 28;12:1562240. doi: 10.3389/fnut.2025.1562240 (PMC12336065; doi:10.3389/fnut.2025.1562240)
Supplement: Supplementary file 1 [file Data_Sheet_1.pdf]

frontiers

SUPPLEMENTARY MATERIAL

DIET PLAN FOR GESTATIONAL DIABETES MELLITUS - SECOND TRIMESTER

VEGETARIAN DIET PLAN

MEAL TIMINGS	FOOD ITEMS	QUANTITY	ENERGY (Kcal)	PROTEIN (g)	CARBOHYDRATES (g)	FAT (g)	FIBRE (g)
EARLY MORNING 06.00 AM – 07.00 AM	Skimmed milk without Sugar /Jaggery/Brown sugar	100ml	29	2.5	4.6	0.1	0
	Nuts - Almonds/Walnuts	6nos (7-25g)	45-169	1.5-3.8	1.6-2.6	3.6-16.2	0.9-1.4
BREAKFAST 08.00 AM – 09.00 AM	Fruits – Apple / Oranges / Pomegranate / Guava or	50g	21-41.5	0.1-1.3	4.5-9.3	0.1-0.6	0.6-2.7
	Vegetable salad - boiled carrots, boiled beans and boiled cauliflower with a pinch of salt and pepper						
	Paneer curry with less oil	100g	105	5.4	3.5	7.8	0.9
	Thick dhal (Moong dal) /Sambar (Thoor dal) or	50g	15-38	0.2-2.5	1.5-6.3	0.1-2.8	0.9-1.7
	Chutney – Ridge gourd / Bottle gourd /Mint chutney/tomato chutney						
	Pongal - Little Millet/Broken Wheat or	100g	74-218	1.8-8.8	11-43.2	1.2-10.8	1.6-8.2
	Upma - Broken Wheat or						
	Kitchadi - Broken wheat / Kodo millet / Little millet or						
	Idiyappam - Finger millet or						
	Idli - Finger millet/ Pearl millet or	2nos (100g)					
	Adai - Foxtail millet/ Pearl millet/ Finger millet/ Sorghum or						
	Dosa - Finger millet/ Pearl millet or						
	Multigrain fenugreek leaves chappati						
MID-MORNING 11.00 AM – 12.00 PM	Fruits – Apple / Oranges / Pomegranate / Guava	100g	48-83	0.2-2.6	10.9-18.7	0.2-1.2	1.1-5.4
	Cucumber	100g	15	0.7	3.6	0.1	0.5
	Buttermilk	100ml	18	0.9	1	1.2	0
LUNCH 01.00 PM – 02.00 PM	Vegetable poriyal/kootu - ladies' finger, green beans, cabbage, green peas, brinjal and cauliflower	75g	68	1.9	8	3.3	2.8
	Greens kootu/poriyal	75g	60	2.5	5.5	3.1	2.1
	Lemon juice (Squeeze 1 tsp lemon juice on top of greens poriyal just before consumption)	1 tsp	1	0	0.4	0	0
	Sambar (Thoor dal)	100ml	76	3.7	108	2	2.5
	Curd	100g	60	3.1	3	4	0
	White rice/brown rice/little millet/ Kodo millet/ Pearl millet (cooked using straining method)	100g	76-103	1.9-3.3	14.3-21.5	0.3-1.4	0.4-3.2
EVENING SNACKS 04.00 PM – 05.00 PM	Steamed Sundals or Steamed sprouts or Steamed sorghum	100g	92-155	2.8-9.9	17.2-26.6	0.5-1.4	2.4-9.6
	Skimmed milk without Sugar /Jaggery/Brown sugar	100ml	29	2.5	4.6	0.1	0
	Vegetable soup - carrots, beans and cauliflower with a pinch of salt and pepper	125ml	42	1.4	6.1	1.5	2
	Vegetable salad - boiled carrots, boiled beans and boiled cauliflower with a pinch of salt and pepper or	50g	21-46	0.9-1.3	4.5-5.4	0.2-2.2	1.2-1.9
	Vegetable poriyal/kootu - ladies' finger, green beans, cabbage, green peas, brinjal and cauliflower						

SUPPLEMENTARY MATERIAL

DINNER 06.30 PM – 07.00 PM	Paneer curry with less oil	100g	105	5.4	3.5	7.8	0.9
	Thick dhal (Moong dal) /Sambar (Thoor dal) or	50g	15-38	0.2-2.5	1.5-6.3	0.1-2.8	0.9-1.7
	Chutney – Ridge gourd / Bottle gourd /Mint chutney/tomato chutney						
	Pongal - Little Millet/Broken Wheat or	100g	74-218	1.8-8.8	11-43.2	1.2-10.8	1.6-8.2
	Upma - Broken Wheat or						
	Kitchadi - Broken wheat / Kodo millet / Little millet or						
	Idiyappam - Finger millet or						
	Idli - Finger millet/ Pearl millet or	2nos (100g)					
	Adai - Foxtail millet/ Pearl millet/ Finger millet/ Sorghum or						
	Dosa - Finger millet/ Pearl millet or						
	Multigrain fenugreek leaves chappati						
BEDTIME 08.30 PM – 09.00 PM	Skimmed milk without Sugar /Jaggery/Brown sugar	100ml	29	2.5	4.6	0.1	0
**TOTAL**			**1118-1746.5**	**43.9-77.3**	**234.4-339.5**	**38.6-81.3**	**23.3-55.7**
**REQ**			**1725**	**65**	**259**	**48**	**28**

NON-VEGETARIAN DIET PLAN

MEAL TIMINGS	FOOD ITEMS	QUANTITY	ENERGY (Kcal)	PROTEIN (g)	CARBOHYDRATES (g)	FAT (g)	FIBRE (g)
EARLY MORNING 06.00 AM – 07.00 AM	Skimmed milk without Sugar /Jaggery/Brown sugar	100ml	29	2.5	4.6	0.1	0
	Nuts - Almonds/Walnuts	6nos (7-25g)	45-169	1.5-3.8	1.6-2.6	3.6-16.2	0.9-1.4
BREAKFAST 08.00 AM – 09.00 AM	Fruits – Apple / Oranges / Pomegranate / Guava or	50g	21-41.5	0.1-1.3	4.5-9.3	0.1-0.6	0.6-2.7
	Vegetable salad - boiled carrots, boiled beans and boiled cauliflower with a pinch of salt and pepper						
	Egg whites omelette with vegetables	2no (126g)	109	7.9	4.7	6.3	1.7
	Thick dhal (Moong dal) /Sambar (Thoor dal) or	50g	15-38	0.2-2.5	1.5-6.3	0.1-2.8	0.9-1.7
	Chutney – Ridge gourd / Bottle gourd /Mint chutney/tomato chutney						
	Pongal - Little Millet/Broken Wheat or	100g	74-218	1.8-8.8	11-43.2	1.2-10.8	1.6-8.2
	Upma - Broken Wheat or						
	Kitchadi - Broken wheat / Kodo millet / Little millet or						
	Idiyappam - Finger millet or						
	Idli - Finger millet/ Pearl millet or	2nos (100g)					
	Adai - Foxtail millet/ Pearl millet/ Finger millet/ Sorghum or						
	Dosa - Finger millet/ Pearl millet or						
	Multigrain fenugreek leaves chappati						
[illegible]	Fruits – Apple / Oranges / Pomegranate / Guava	100g	48-83	0.2-2.6	10.9-18.7	0.2-1.2	1.1-5.4

SUPPLEMENTARY MATERIAL

11.00 AM – 12.00 PM	Cucumber	100g	15	0.7	3.6	0.1	0.5
	Buttermilk	100ml	18	0.9	1	1.2	0
	Vegetable poriyal/kootu - ladies' finger, green beans, cabbage, green peas, brinjal and cauliflower	100g	91	2.5	10.7	4.4	3.7
	Greens kootu/poriyal	150g	120	5	11	6.2	4.1
	Lemon juice (Squeeze 1 tsp lemon juice on top of greens poriyal just before consumption)	1 tsp	1	0	0.4	0	0
**LUNCH 01.00 PM – 02.00 PM**							
	Sambar (Thoor dal) or	100ml	76-105	1.1-11.4	3.5-10.8	2-6.8	1.2-2.6
	Fish curry (TO AVOID - shark, swordfish, king mackerel, tile fish because of high mercury content) or						
	Chicken breast curry						
	Steamed fish (TO AVOID - shark, swordfish, king mackerel, tile fish because of high mercury content) or	1no (80g)	17-74	3.6-12.7	0.2-3.4	0.2-1.1	0
	Boiled egg white	1no (32g)					
	White rice/brown rice/little millet/ Kodo millet/ Pearl millet (cooked using straining method)	100g	76-103	1.9-3.3	14.3-21.5	0.3-1.4	0.4-3.2
**EVENING SNACKS 04.00 PM – 05.00 PM**	Steamed Sundals or Steamed sprouts or Steamed sorghum	100g	92-155	2.8-9.9	17.2-26.6	0.5-1.4	2.4-9.6
	Skimmed milk without Sugar /Jaggery/Brown sugar	100ml	29	2.5	4.6	0.1	0
**DINNER 06.30 PM – 07.00 PM**	Vegetable soup - carrots, beans and cauliflower with a pinch of salt and pepper	125ml	42	1.4	6.1	1.5	2
	Vegetable salad - boiled carrots, boiled beans and boiled cauliflower with a pinch of salt and pepper or	100g	91-134	2.5-4.3	10.7-11.4	4.4-8	2.5-3.7
	Vegetable poriyal/kootu - ladies' finger, green beans, cabbage, green peas, brinjal and cauliflower						
	Egg whites omelette with vegetables	2no (126g)	109	7.9	4.7	6.3	1.7
	Thick dhal (Moong dal) /Sambar (Thoor dal) or	50g	15-38	0.2-2.5	1.5-6.3	0.1-2.8	0.9-1.7
	Chutney – Ridge gourd / Bottle gourd /Mint chutney/tomato chutney						
	Pongal - Little Millet/Broken Wheat or	100g	74-218	1.8-8.8	11-43.2	1.2-10.8	1.6-8.2
	Upma - Broken Wheat or						
	Kitchadi - Broken wheat / Kodo millet / Little millet or						
	Idiyappam - Finger millet or						
	Idli - Finger millet/ Pearl millet or	2nos (100g)					
	Adai - Foxtail millet/ Pearl millet/ Finger millet/ Sorghum or						
	Dosa - Finger millet/ Pearl millet or						
	Multigrain fenugreek leaves chappati						
**BEDTIME 08.30 PM – 09.00 PM**	Skimmed milk without Sugar /Jaggery/Brown sugar	100ml	29	2.5	4.6	0.1	0
**TOTAL**			**1236-1968.5**	**51.5-105.7**	**143.9-259.3**	**40.2-90.2**	**27.9-62.2**
**REQ**			**1725**	**65**	**259**	**48**	**28**

SUPPLEMENTARY MATERIAL

DIET PLAN FOR GESTATIONAL DIABETES MELLITUS - THIRD TRIMESTER							
**VEGETARIAN DIET PLAN**							
**MEAL TIMINGS**	**FOOD ITEMS**	**QUANTITY**	**ENERGY (Kcal)**	**PROTEIN (g)**	**CARBOHYDRATES (g)**	**FAT (g)**	**FIBRE (g)**
**EARLY MORNING 06.00 AM – 07.00 AM**	Skimmed milk without Sugar /Jaggery/Brown sugar	100ml	29	2.5	4.6	0.1	0
	Nuts - Almonds/Walnuts	6nos (7-25g)	45-169	1.5-3.8	1.6-2.6	3.6-16.2	0.9-1.4
**BREAKFAST 08.00 AM – 09.00 AM**	Fruits – Apple / Oranges / Pomegranate / Guava or	50g	21-41.5	0.1-1.3	4.5-9.3	0.1-0.6	0.6-2.7
	Vegetable salad - boiled carrots, boiled beans and boiled cauliflower with a pinch of salt and pepper						
	Paneer curry with less oil	100g	105	5.4	3.5	7.8	0.9
	Thick dhal (Moong dal) /Sambar (Thoor dal) or	50g	15-38	0.2-2.5	1.5-6.3	0.1-2.8	0.9-1.7
	Chutney – Ridge gourd / Bottle gourd /Mint chutney/tomato chutney						
	Pongal - Little Millet/Broken Wheat or	100g	74-218	1.8-8.8	11-43.2	1.2-10.8	1.6-8.2
	Upma - Broken Wheat or						
	Kitchadi - Broken wheat / Kodo millet / Little millet or						
	Idiyappam - Finger millet or						
	Idli - Finger millet/ Pearl millet or	2nos (100g)					
	Adai - Foxtail millet/ Pearl millet/ Finger millet/ Sorghum or						
	Dosa - Finger millet/ Pearl millet or						
	Multigrain fenugreek leaves chappati						
**MID-MORNING 11.00 AM – 12.00 PM**	Fruits – Apple / Oranges / Pomegranate / Guava	100g	48-83	0.2-2.6	10.9-18.7	0.2-1.2	1.1-5.4
	Cucumber	100g	15	0.7	3.6	0.1	0.5
	Buttermilk	100ml	18	0.9	1	1.2	0
**LUNCH 01.00 PM – 02.00 PM**	Vegetable poriyal/kootu - ladies' finger, green beans, cabbage, green peas, brinjal and cauliflower	75g	68	1.9	8	3.3	2.8
	Greens kootu/poriyal	75g	60	2.5	5.5	3.1	2.1
	Lemon juice (Squeeze 1 tsp lemon juice on top of greens poriyal just before consumption)	1 tsp	1	0	0.4	0	0
	Sambar (Thoor dal)	100ml	76	3.7	108	2	2.5
	Curd	100g	60	3.1	3	4	0
	White rice/brown rice/little millet/ Kodo millet/ Pearl millet (cooked using straining method)	100g	76-103	1.9-3.3	14.3-21.5	0.3-1.4	0.4-3.2
**EVENING SNACKS 04.00 PM – 05.00 PM**	Steamed Sundals or Steamed sprouts or Steamed sorghum	100g	92-155	2.8-9.9	17.2-26.6	0.5-1.4	2.4-9.6
	Skimmed milk without Sugar /Jaggery/Brown sugar	100ml	29	2.5	4.6	0.1	0

	Vegetable salad - boiled carrots, boiled beans and boiled cauliflower with a pinch of salt and pepper or	75g	27-68	0.7-1.9	4.9-8	0.7-3.3	1.6-2.8
	Vegetable poriyal/kootu - ladies' finger, green beans, cabbage, green peas, brinjal and cauliflower						
	Paneer curry with less oil	100g	105	5.4	3.5	7.8	0.9
	Thick dhal (Moong dal) /Sambar (Thoor dal) or	50g	15-38	0.2-2.5	1.5-6.3	0.1-2.8	0.9-1.7
	Chutney – Ridge gourd / Bottle gourd /Mint chutney/tomato chutney						
**DINNER 06.30 PM – 07.00 PM**							
	Pongal - Little Millet/Broken Wheat or	100g	74-218	1.8-8.8	11-43.2	1.2-10.8	1.6-8.2
	Upma - Broken Wheat or						
	Kitchadi - Broken wheat / Kodo millet / Little millet or						
	Idiyappam - Finger millet or						
	Idli - Finger millet/ Pearl millet or	2nos (100g)					
	Adai - Foxtail millet/ Pearl millet/ Finger millet/ Sorghum or						
	Dosa - Finger millet/ Pearl millet or						
	Multigrain fenugreek leaves chappati						
**BEDTIME 08.30 PM – 09.00 PM**	Skimmed milk without Sugar /Jaggery/Brown sugar	100ml	29	2.5	4.6	0.1	0
**TOTAL**			**1082-1726.5**	**42.3-77**	**228.7-336**	**37.6-80.9**	**21.7-54.6**
**REQ**			**1725**	**77**	**259**	**42**	**28**

NON-VEGETARIAN DIET PLAN

MEAL TIMINGS	FOOD ITEMS	QUANTITY	ENERGY (Kcal)	PROTEIN (g)	CARBOHYDRATES (g)	FAT (g)	FIBRE (g)
**EARLY MORNING 06.00 AM – 07.00 AM**	Skimmed milk without Sugar /Jaggery/Brown sugar	100ml	29	2.5	4.6	0.1	0
	Nuts - Almonds/Walnuts	6nos (7-25g)	45-169	1.5-3.8	1.6-2.6	3.6-16.2	0.9-1.4
**BREAKFAST 08.00 AM – 09.00 AM**	Fruits – Apple / Oranges / Pomegranate / Guava or	50g	21-41.5	0.1-1.3	4.5-9.3	0.1-0.6	0.6-2.7
	Vegetable salad - boiled carrots, boiled beans and boiled cauliflower with a pinch of salt and pepper						
	Egg whites omelette with vegetables	2no (126g)	109	7.9	4.7	6.3	1.7
	Thick dhal (Moong dal) /Sambar (Thoor dal) or	50g	15-38	0.2-2.5	1.5-6.3	0.1-2.8	0.9-1.7
	Chutney – Ridge gourd / Bottle gourd /Mint chutney/tomato chutney						
	Pongal - Little Millet/Broken Wheat or	100g	74-218	1.8-8.8	11-43.2	1.2-10.8	1.6-8.2
	Upma - Broken Wheat or						
	Kitchadi - Broken wheat / Kodo millet / Little millet or						
	Idiyappam - Finger millet or						
	Idli - Finger millet/ Pearl millet or						
	Adai - Foxtail millet/ Pearl millet/ Finger millet/ Sorghum or						

	Dosa - Finger millet/ Pearl millet or	2nos (100g)					
	Multigrain fenugreek leaves chappati						
**MID-MORNING 11.00 AM – 12.00 PM**	Fruits – Apple / Oranges / Pomegranate / Guava	100g	48-83	0.2-2.6	10.9-18.7	0.2-1.2	1.1-5.4
	Cucumber	100g	15	0.7	3.6	0.1	0.5
	Buttermilk	125ml	23	1.2	1.2	1.5	0
	Greens kootu/poriyal	150g	120	5	11	6.2	4.1
	Vegetable poriyal/kootu - ladies' finger, green beans, cabbage, green peas, brinjal and cauliflower	100g	91	2.5	10.7	4.4	3.7

**LUNCH 01.00 PM – 02.00 PM**	Lemon juice (Squeeze 1 tsp lemon juice on top of greens poriyal just before consumption)	1 tsp	1	0	0.4	0	0
	Sambar (Thoor dal) or	100ml	76-105	1.1-11.4	3.5-10.8	2-6.8	1.2-2.6
	Fish curry (TO AVOID - shark, swordfish, king mackerel, tile fish because of high mercury content) or						
	Chicken breast curry						
	Steamed fish (TO AVOID - shark, swordfish, king mackerel, tile fish because of high mercury content) or	1no (80g)	17-74	3.6-12.7	0.2-3.4	0.2-1.1	0
	Boiled egg white	1no (32g)					
	White rice/brown rice/little millet/ Kodo millet/ Pearl millet (cooked using straining method)	100g	76-103	1.9-3.3	14.3-21.5	0.3-1.4	0.4-3.2
**EVENING SNACKS 04.00 PM – 05.00 PM**	Steamed Sundals or Steamed sprouts or Steamed sorghum	100g	92-155	2.8-9.9	17.2-26.6	0.5-1.4	2.4-9.6
	Skimmed milk without Sugar /Jaggery/Brown sugar	100ml	29	2.5	4.6	0.1	0
**DINNER 06.30 PM – 07.00 PM**	Vegetable soup - carrots, beans and cauliflower with a pinch of salt and pepper	125ml	42	1.4	6.1	1.5	2
	Vegetable salad - boiled carrots, boiled beans and boiled cauliflower with a pinch of salt and pepper or	100g	91-134	2.5-4.3	10.7-11.4	4.4-8	2.5-3.7
	Vegetable poriyal/kootu - ladies' finger, green beans, cabbage, green peas, brinjal and cauliflower						
	Egg whites omelette with vegetables	2no (126g)	109	7.9	4.7	6.3	1.7
	Thick dhal (Moong dal) /Sambar (Thoor dal) or	50g	15-38	0.2-2.5	1.5-6.3	0.1-2.8	0.9-1.7
	Chutney – Ridge gourd / Bottle gourd /Mint chutney/tomato chutney						
	Pongal - Little Millet/Broken Wheat or	100g	74-218	1.8-8.8	11-43.2	1.2-10.8	1.6-8.2
	Upma - Broken Wheat or						
	Kitchadi - Broken wheat / Kodo millet / Little millet or						
	Idiyappam - Finger millet or						
	Idli - Finger millet/ Pearl millet or	2nos (100g)					
	Adai - Foxtail millet/ Pearl millet/ Finger millet/ Sorghum or						
	Dosa - Finger millet/ Pearl millet or						
	Multigrain fenugreek leaves chappati						
**BEDTIME 08.30 PM – 09.00 PM**	Skimmed milk without Sugar /Jaggery/Brown sugar	100ml	29	2.5	4.6	0.1	0

SUPPLEMENTARY MATERIAL

TOTAL			1241-1973.5	51.8-106	144.1-259.5	40.5-90.5	27.8-62.1
REQ			1725	77	259	42	28
